# Crystal structure and biochemical characterization of Chlamydomona*s* FDX2 reveal two residues that, when mutated, partially confer FDX2 the redox potential and catalytic properties of FDX1

**DOI:** 10.1007/s11120-015-0198-6

**Published:** 2015-11-03

**Authors:** Marko Boehm, Markus Alahuhta, David W. Mulder, Erin A. Peden, Hai Long, Roman Brunecky, Vladimir V. Lunin, Paul W. King, Maria L. Ghirardi, Alexandra Dubini

**Affiliations:** Biosciences Center, National Renewable Energy Laboratory, Mail Stop: 3313, 15013 Denver West Parkway, Golden, CO 80401 USA; Computational Science Center, National Renewable Energy Laboratory, 15013 Denver West Parkway, Golden, CO 80401 USA

**Keywords:** Ferredoxin, Chlamydomonas, Structure, Interaction, NADPH, Hydrogen photo-production

## Abstract

The green alga *Chlamydomonas reinhardtii* contains six plastidic [2Fe2S]-cluster ferredoxins (FDXs), with FDX1 as the predominant isoform under photoautotrophic growth. FDX2 is highly similar to FDX1 and has been shown to interact with specific enzymes (such as nitrite reductase), as well as to share interactors with FDX1, such as the hydrogenases (HYDA), ferredoxin:NAD(P) reductase I (FNR1), and pyruvate:ferredoxin oxidoreductase (PFR1), albeit performing at low catalytic rates. Here we report the FDX2 crystal structure solved at 1.18 Å resolution. Based on differences between the *Chlorella fusca* FDX1 and *C. reinhardtii* FDX2 structures, we generated and purified point-mutated versions of the FDX2 protein and assayed them in vitro for their ability to catalyze hydrogen and NADPH photo-production. The data show that structural differences at two amino acid positions contribute to functional differences between FDX1 and FDX2, suggesting that FDX2 might have evolved from FDX1 toward a different physiological role in the cell. Moreover, we demonstrate that the mutations affect both the midpoint potentials of the FDX and kinetics of the FNR reaction, possibly due to altered binding between FDX and FNR. An effect on H_2_ photo-production rates was also observed, although the kinetics of the reaction were not further characterized.

**Electronic supplementary material:**

The online version of this article (doi:10.1007/s11120-015-0198-6) contains supplementary material, which is available to authorized users.

## Introduction

Ferredoxins (FDXs) are small, ubiquitous proteins that typically contain iron-sulfur clusters and mediate electron shuttling among multiple metabolic pathways. The green alga *Chlamydomonas reinhardtii* (Chlamydomonas throughout) contains six genes that encode for chloroplast-localized ferredoxins. These proteins have been categorized into three separate groups, according to the plant nomenclature: FDX1 and FDX5 belong to the photosynthetic category (or leaf type), FDX2 belong to the non-photosynthetic group (or root type), and FDX3, FDX4, and FDX6 are divergent, being more closely related to bacterial FDXs (Terauchi et al. 2009). In their oxidized state, FDXs display absorption maxima in the visible region at about 330, 420, and 464 nm, and their redox potentials are very negative; FDX1, for example, is centered at around −398 mV (Wada et al. 1974; Orme-Johnson 1973; Matsubara and Sasaki 1968; Hutson et al. 1978; Hase et al. 1976). FDXs harbor a CX_4_CX_2_CX_n_C motif required for [2Fe2S]-cluster ligation and form specific electrostatic complexes with a variety of interacting enzymes, using carboxyl side-chain groups of conserved amino acids that generally interact with lysine or arginine counterparts on the binding partner.

As electron shuttles, Chlamydomonas FDXs are particularly important for hydrogen production, because they are the natural electron donor to the hydrogenases in vivo. Chlamydomonas contains two [FeFe]-hydrogenase enzymes, *Cr*HYDA1 and *Cr*HYDA2, which catalyze the formation of hydrogen from two electrons and two protons, either under light-anoxic or dark-anoxic conditions (Ghirardi et al. 2000; Harris 2009). FDXs represent a branch point for three hydrogen production pathways (Meuser et al. 2012; Winkler et al. 2010): the PSII dependent, PSII independent, and fermentative.

To further characterize the role of the algal FDXs, Peden et al. (2013) identified specific targets for each of the six Chlamydomonas FDXs and assessed *Cr*FDX1 and *Cr*FDX2 specificity toward selected metabolic pathways. Using yeast two-hybrid and pull-down assays, they detected binding partners for the two *Cr*FDXs and confirmed that *Cr*FDX2 can also interact with common *Cr*FDX1 interaction partners. *Cr*FDX2 amino acid sequence is highly homologous to that of *Cr*FDX1 (67 % identity for the mature proteins), and it contains conserved residues that are known to be important for interactions with *Cr*FDX1 enzyme targets (Winkler et al. 2009b). This sequence conservation might explain why *Cr*FDX2 participates in electron-transfer reactions with similar redox partners as *Cr*FDX1, although at lower rates. Both proteins contain three highly conserved, negatively charged, solvent-exposed regions that were proposed to be responsible for mediating protein–protein interactions in FDXs (Kameda et al. 2011). Typically, these regions form a highly conserved structure that facilitates cluster insertion to the apo-protein and electron transfer to/from the mature protein (Bertini et al. 2002; Kameda et al. 2011). Four conserved amino acid residues present in the two *Cr*FDXs specifically contribute to electron donor/acceptor selectivity in vivo (Terauchi et al. 2009). Indeed, *Cr*FDX2 has recently been shown to mediate electron transfer (although not as efficiently as *Cr*FDX1) to known *Cr*FDX1 interaction partners, i.e., *Cr*FNR1, *Cr*HYDA1, and *Cr*PFR1, (Noth et al. 2012; Peden et al. 2013; Terauchi et al. 2009; van Lis et al. 2013). However, it is unknown whether or under which conditions this happens in vivo.

Structural models have predicted differences in surface charge distribution on the two *Cr*FDXs, which may explain *Cr*FDX1′s more negative redox potential (−398 vs −321 mV for *Cr*FDX2) (Galván and Márquez 1985; Terauchi et al. 2009). These distinct physical characteristics might determine their interaction specificity and influence the binding of various electron acceptors/donors to each protein (Terauchi et al. 2009). Notably, *Cr*FDX2 lacks a phenylalanine (F62) residue that may be required for the proper interaction of *Cr*FDX1 with *Cr*HYDA (Winkler et al. 2009a) and possibly with *Cr*FNR1 (Hurley et al. 1997; Mayoral et al. 2005); it also lacks a C-terminus tyrosine residue, Y95, present in *Cr*FDX1. The lower capability of *Cr*HYDA1 to generate hydrogen using *Cr*FDX2 as the electron donor might be indicative not only of their different redox potentials but also of different interaction mechanisms between *Cr*HYDA1 and either *Cr*FDX1 or *Cr*FDX2.

In an effort to better define these interactions and the function of *Cr*FDX1 and *Cr*FDX2, we over-expressed the two proteins in *E. coli*. The mature versions of these proteins were purified, used for crystallography studies, and characterized by different types of spectroscopic techniques (see supplemental data 1, 2, 3, and 4). Here we report the first 3D structure of a Chlamydomonas ferredoxin, *Cr*FDX2, at atomic resolution of 1.18 Å. The *Cr*FDX2 folding motif is similar to that of previously published plant-type FDX structures from other organism (Bes et al. 1999; Fish et al. 2005), one of which represents a FDX1 type from *Chlorella fusca* (*Cf*), with high sequence homology to the noncrystallized *Cr*FDX1. Based on the high degree of similarity between *Cf* and *Cr* FDX1, comparison with the *Cr* FDX2 structure, and published data, we selected two amino acid residues present on the interaction surface of *Cr*FDX1 with FNR and hydrogenase (but absent or present as a different residue on *Cr*FDX2). We mutated *Cr*FDX2 F62 to M62 (the equivalent residue in *Cr*FDX1) and inserted Y95 into *Cr*FDX2 through site-directed mutagenesis. We show that mutations that replace these residues with those found in *Cr*FDX1 lower *Cr*FDX2′s midpoint redox potential to values closer to that of *Cr*FDX1, indicating that these residues contribute to functional differences between *Cr*FDX1 and *Cr*FDX2. The major observed difference consisted of altered midpoint redox potential of the FDX cluster, resulting in changes in its catalytic efficiency with respect to FNR-mediated NADP^+^ reduction, as well as alterations in the maximum rates of hydrogenase-catalyzed H_2_ photo-production in vitro.

## Materials and methods

### Plasmid construction

We constructed several over-expression plasmids for all the mature, codon-optimized (Mr. Gene, Germany) versions of the Chlamydomonas FDX proteins, using a modified version of the pRSETA vector (Life Technologies, USA; Michoux et al. 2010) as the backbone. For the pull-down experiments, we used N-terminal His-tagged *Cr*FDX1 and *Cr*FDX2 proteins that were expressed from the pRESTA His-FDX1 and pRESTA His-FDX 2 expression constructs, respectively. For this, the *Cr*FDX1 and *Cr*FDX2 coding sequences were amplified using FDX1 primers (Peden et al. 2013) and the following FDX2 primers: FDX2-Fw GGATCCTTCAAAGTCACCTTCAAAACCCCAAAAGGTG-3′ and FDX2-Rev 5′-ACATCGTCATTTTAACCGATCAAGAATCAAAATTGTGAGAATTC-3. The amplified gene sequences were cloned into the BamHI and EcoRI restriction sites on the vector. The tobacco etch virus protease (TEV)-cleaved versions of the *Cr*FDX1 and *Cr*FDX2 proteins were expressed as His-GST-TEVcs-FDX1 and His-GST-TEVcs-FDX2 fusion proteins and contained a linker sequence (Yacoby et al. 2012) between the His-GST tandem affinity tag and the FDX sequence. These versions were used for UV/Vis and EPR spectroscopy. The cleaved versions of the *Cr*FDX1 and *Cr*FDX2 proteins used for CD spectroscopy that yielded *Cr*FDX2 crystals for X-ray crystallography were expressed as FDX1-TEVcs-GST-His and FDX2-TEVcs-GST-His fusion proteins that contained the same linker sequence (Yacoby et al. 2012) between the FDX and the GST-His tandem affinity tag. To generate the expression constructs for the point-mutated *Cr*FDX2 s (M62F, ∇95Y and M62F/∇95Y), the C-terminal-encoding fragment of the *Cr*FDX2 protein was excised from the His-GST-TEVcs-FDX2 construct using HincII and EcoRI. Subsequently, digested PCR fragments generated with the following primers replaced the excised fragment: (A) *Cr*FDX2 M62F: Fw 5′-GCGGTCGACCAATCCGACCAAAACTTTTTGGACGAAGATCAATTG-3′, (B) *Cr*FDX2 M62F Rv: 5′-GGAATTCTCACAATTTTGATTCTTGATC.

GGTTAAAATGACGATGT-3′, (C) *Cr*FDX2 ∇Y95 Fw: 5′-GCGTACTGTCGACCAATCCGACCAA.

AACATG-3′, and (D) *Cr*FDX2 ∇Y95 Rev: 5′-GAATTCTCAGTACAATTTTGATTCTTGATCGGTTAAAATGACGATGT-3′. The appropriate primer combinations were used to introduce the two point mutations into the WT FDX2.

### Protein purification

The *Cr*FDX1 and *Cr*FDX2 over-expression plasmids were transformed into *E. coli* KRX cells (Promega, USA). For expression in Terrific Broth with 200 μg/ml Ampicillin (TB; VWR, USA), a starter culture was grown overnight at 37 °C and diluted 1:100 in a 100-ml TB subculture the following morning. After the subculture had reached an OD600 of ~0.7, 10 ml of it were used to inoculate 1 L of TB media supplemented with 0.4 % (w/v) glycerol. At OD600 of ~0.7, IPTG and Ferric ammonium citrate were added to final concentrations of 1 mM and 0.05 % (w/v), respectively, (Peden et al. 2013). The cells were harvested and resuspended in 100 ml lysis buffer (25 mM Tris pH 7.9, 100 mM NaCl, and 1 mM DTT) for breakage. The supernatant obtained after centrifugation was incubated for 1 h at 4 °C with 20 ml of glutathione affinity resin (Genscript, USA). After the incubation period, the resin was washed with 15 column volumes (CV) of lysis buffer or until the wash solution became clear and colorless. Protein elution was performed with 2 × CV of elution buffer (25 mM Tris pH = 7.9, 100 mM NaCl, and 10 mM reduced glutathione). 20 mg of TEV-His (His-tag purified from pRK193 (Kapust et al. 2001; Addgene, USA) were added to cleave the affinity tag. After a 2-h incubation period at RT, the sample was applied to a TALON Cobalt affinity chromatography column (~20 ml resin (Clontech, USA) packed in a XK16/20 (GE Healthcare column) coupled to an Äkta FPLC (GE Healthcare, USA), see supplemental Fig. 1. We used 25 mM Tris pH 7.0, 100 mM NaCl, and 5 % (v/v) glycerol as the running buffer at a flow rate of 5–10 ml min^−1^. The flow-through was collected and loaded onto a HiLoad™ 26/60 Superdex™ 75 prep grade (GE Healthcare, USA) following the purification method developed by Peden et al. (2013). The iron content was determined using a colorimetric assay described (Winkler et al. 2009b), which uses ferrozine under reductive conditions after digestion of the protein in 4.5 % (w/v) KMnO4 and 1.2 N HCl.

### Protein crystallization

*Cr*FDX2 protein crystals were obtained by the sitting drop vapor diffusion method, using a 96-well plate with Crystal Screen HT (Hampton Research, USA). Fifty µL of well solution were added to the reservoirs and drops were made with 0.2 µL of well and 0.2 µL of protein solution using a Phoenix crystallization robot (Art Robbins Instruments, USA). Protein crystals grew at 20 °C in 0.1 M HEPES pH 7.0 and 3.2 M ammonium sulfate as the well solution. The protein solution contained 17.3 mg/ml of protein in 25 mM Tris pH 7.0, 200 mM NaCl, and 5 % (v/v) glycerol.

### Data collection and processing

The *Cr*FDX2 protein crystal was flash-frozen in a nitrogen gas stream at 100 K before data collection, using an in-house Bruker × 8 MicroStar X-Ray generator with Helios mirrors and Bruker Platinum 135 CCD detector. A well solution containing 5 % (v/v) glycerol and 5 % (v/v) ethylene glycol was added into the drop before freezing to prevent ice formation. Data were indexed and processed with the Bruker suite of programs version 2011.2-0 (Bruker AXS, USA).

### Structure solution and refinement

**Fig. 1:**
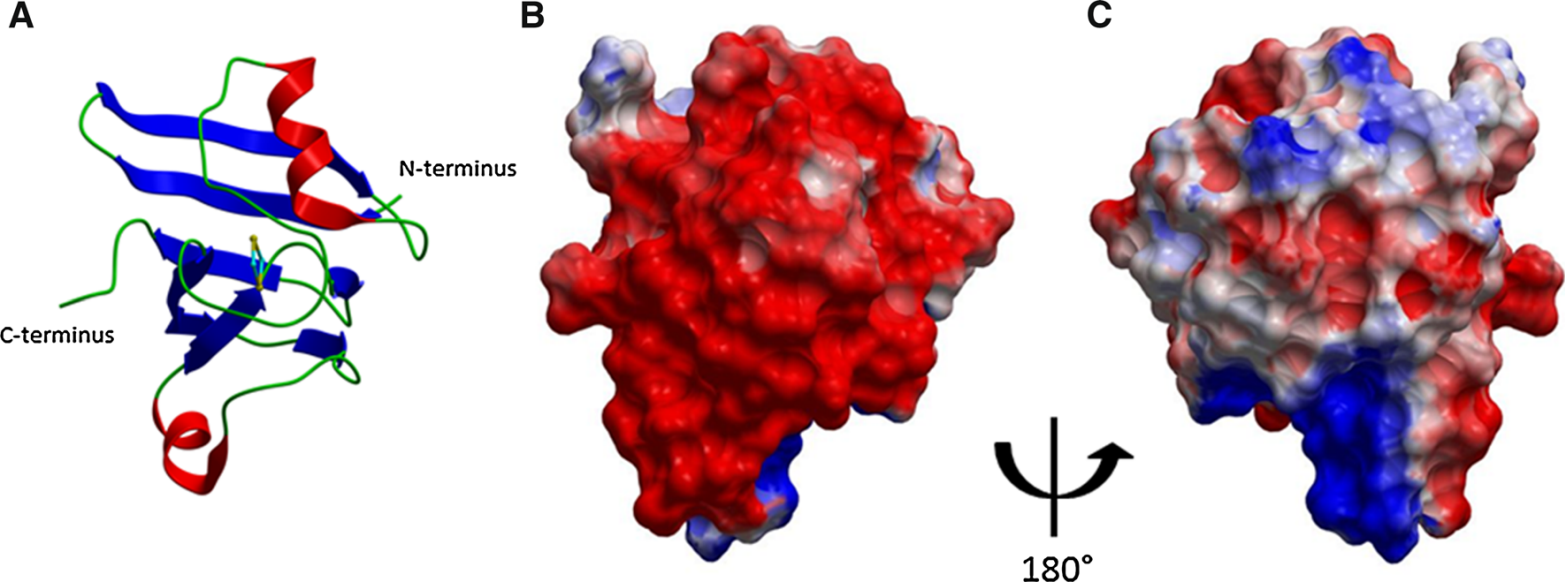
*Cr*FDX2 secondary structure and surface charge distribution models. **a** Ribbon diagram of the *Cr*FDX2 structure with the [2Fe2S] cluster. N- and C-termini are indicated. Loops are shown as *green* ribbons; α-helices are in *red* and β-strands in *blue*; the [2Fe2S] cluster is shown in ball and stick format, with sulfur in yellow and iron atoms in cyan. **b**, **c** Electrostatic surfaces charges are shown in *red* (negative) and *blue* (positive). *Panel B* is shown in the same orientation as *Panel A* and represents a straight-on view of the [2Fe2S] cluster. *Panel C* is rotated 180° with respect to *panels A* and *B*. The orientation chosen here highlights the different-charged region of *Cr*FDX2 with the *red region* being the area of *Cr*FNR/*Cr*HYDA1 interaction

**Fig. 2:**
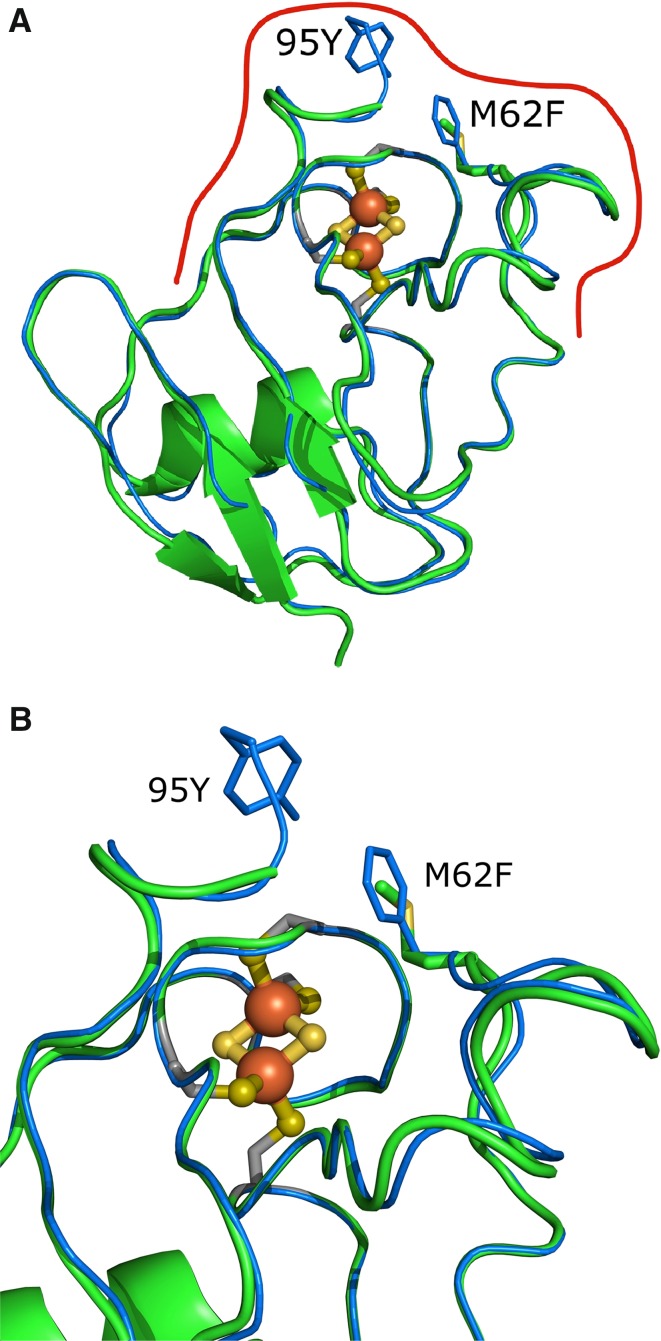
*Cr*FDX2 binding interface with *Cr*HYDA1/*Cr*FNR1 and point mutations. **a** Overall view of *Cr*FDX2 superimposed with *Cf*FDX1. Mutations and the approximate binding interface area indicated by a *red line*. **b** Closer view of the mutations. *Coloring* The backbone of *Cr*FDX2 is shown as *green* ribbon and *Cf*FDX1 backbone is shown in *blue*; oxygen atoms are *red*, nitrogen atoms are *blue*, sulfur atoms are *yellow*, and the coordinating cysteine side-chain carbons are *colored gray*. The [2Fe2S] cluster is shown in ball and stick, with sulfur in *yellow* and iron in *orange*. The main chains of the residue stick representations have been hidden for clarity

Intensities obtained from data processing (derived from diffraction intensities) were converted into structure factors and 5 % of the reflections were flagged for Rfree calculations using the programs SCALEPACK2MTZ, ctruncate, MTZDUMP, Unique, CAD, FREERFLAG, and MTZUTILS from the CCP4 package (Winn et al. 2011). The program MrBUMP version 0.6.1 (Winn et al. 2011) automatically solved the structure using the FASTA (Pearson and Lipman 1988) and MOLREP (Vagin and Teplyakov 2010) programs for sequence searches and molecular replacement. Refinement and manual correction were performed using the REFMAC5 (Murshudov et al. 2011) version 5.7.0032 and the Coot (Emsley et al. 2010) version 0.6.2 programs. The MOLPROBITY method (Chen et al. 2010a) was used to analyze the Ramachandran plot, and root mean square deviations (rmsd) of bond lengths and angles were calculated from ideal values of Engh and Huber stereochemical parameters (Engh and Huber 1991). The Wilson B-factor was calculated using the ctruncate version 1.5.1, and average B-factors were calculated using the ICM version 3.7-2a program (Molsoft LLC, USA). Coot, PyMOL (http://www.pymol.org), and ICM (http://www.molsoft.com) were used for comparing and analyzing structures. Figure 1 was done using ICM and PyMOL was used to make Fig. 2. The data collection and refinement statistics are shown in Table 1.

**Table 1:** X-ray data collection and refinement statistics for *Cr*FDX2 crystal structure

Data collection	
Space group	P1
Unit cell (Å, °)	*a* = 25.429, *b* = 26.458, *c* = 31.016 *α* = 102.56, *β* = 104.35, *γ* = 100.30
Wavelength (Å)	1.54178
Temperature (K)	100
Resolution (Å)	25–1.18 (1.28–1.18)
Unique reflections	24232 (5195)
R_int_^a^	0.0838 (0.2948)
Average redundancy	6.1 (2.3)
<I>/<σ(I)>	11.9 (2.6)
Completeness (%)	99.4 (98.1)
Resolution (Å)	25–1.18 (1.21–1.18)
R/R_free_	0.109 (0.246)/0.147 (0.305)
Protein atoms	
Water molecules	
Other atoms	
RMSD from ideal bond length (Å)^b^	0.024
RMSD from ideal bond angles (°)^b^	2.497
Wilson B-factor	
Average B-factor for protein atoms (Å^2^)	
Average B-factor for water molecules (Å^2^)	
Ramachandran plot statistics (%)^c^	
Allowed	100
Favored	98.7
Outliers	0

Statistics for the highest resolution bin are in parenthesis

^a^ ${\text{Rint}} = \sum \left| {I - < I > } \right|/\sum \left| I \right|$ where *I* is the intensity of an individual reflection and <*I*> is the mean intensity of a group of equivalents, and the sums are calculated over all reflections with more than one equivalent measured

^b^Chen et al. (2010b)

^c^Chen et al. (2010a)

### Isolation of thylakoids

In order to generate the thylakoids membrane for the H_2_ and NADP assays, Chlamydomonas cells were harvested from cultures grown in Tris/Acetate/Phosphate medium (TAP) (pH 7.2) (Harris 2009). Algal cultures were maintained at 25 °C, vigorously bubbled with air enriched with 3 % (v/v) CO_2_, stirred using a magnetic stirrer bar, and illuminated with continuous light of 80 μmol photon m^−2^ s^−1^. These cells were then washed with 1/5 volume of buffer 1 (containing 1 0.35 M sorbitol, 20 mM HEPES, pH 7.5, 2.0 mM MgCl2) and repellet as above. The cells were broken using a french press and the thylakoids were pelleted at 40,000 × G (for 20 min at 4 °C). The final thylakoids were resuspended in equal or greater volume of buffer 1, homogenized and spinned at 1200 × G for 30 s to pellet unbroken cells. The supernatant was removed and the pelletted thylakoids stored at −80 °C at with a final concentration of 1.0 mg Chl^−1^ ml^−1^.

### Hydrogen photo-production

A master mix was prepared for the hydrogen photo-production assay as follows (amount per assay): 900 ml buffer A (50 mM tris–HCl pH 7.4, 3.35 mg ml^−1^ bovine serum albumin, 10 mM MgCl_2_, and 200 mg ml^−1^ sucrose), 5 μL of DCPIP (0.01 mM in buffer A), 10 μL of DCMU (0.3 mM in DMSO), 10 μL of sodium ascorbate (1 M), 10 µL glucose oxidase (30 mg/ml in buffer A), 50 µL glucose (1 M), 10 µL catalase (10 mg ml^−1^ in buffer A), 50 µL 96 % (v/v) ethanol, and 100 nM HYDA1 hydrogenase (in buffer A), as described previously (24). All solutions had been previously degassed and were mixed inside a MBRAUN glove box in a 100 % N_2_ atmosphere. FDX was placed in 9-ml serum vials containing the master mix to a final concentration of 10 mM. After addition of Chlamydomonas thylakoids to a final concentration of 25 mg ml^−1^ chlorophyll in the dark, 1.2 ml of the master mix were transferred to serum vials. The vials were sealed with rubber septa and wrapped in aluminum foil. A zero time-point sample was taken, the vial was unwrapped, and illuminated at 400 μmol photons m^−2^ s^−1^ generated by a LED light source (2000 W Diamond Series, www.advancedLEDlights.com). Hydrogen in the head-space was measured at different time points by a gas chromatograph (400 µl injection volume), and the resulting hydrogen production rates were calculated using data from three replicate samples for each FDX tested (μmol H_2_ μg Chl^−1^ h^−1^).

### NADPH photo-production

Initially, three solutions were prepared: (a) a buffer master mix containing (for each FDX/concentration combination) 1 μl of DCMU (0.3 mM in DMSO), 2.5 μl of DCPIP (0.01 mM), 5 μl of sodium ascorbate (1 M), Chlamydomonas thylakoids to a Chl concentration of 50 μg ml^−1^ (in the final assay) and buffer A (see above) to a total volume of 268 μl per buffer master mix; (b) a protein mix (final volume of 208 μl) containing 0.5 μM Chlamydomonas FNR1 (expressed in and purified from *E. coli*) and various concentrations of FDXs (FDX1, FDX2, FDX2 M62F, FDX2 ∇95Y, and FDX2 M62F/∇95Y), and (c) a 0.04 mM NADP^+^ solution. For each assay, 8 μl of the NADP^+^ solution were placed in a well of a 96-well plate. Then, in the dark, 208 μl of the buffer master mix were added to each protein mix and 130 μl of this mixture were added to three separate wells (triplicate samples). The plate was kept in the dark (*t* = 0 was taken) and then illuminated at 300 μmol photons m^−2^ s^−1^ from an LED light source (2000 W Diamond Series, www.advancedLEDlights.com). Further absorbance measurements were taken at various time points and recorded by the Infinite M200Pro plate reader (Tecan, USA). NADPH production rates (μmol NADPH μg Chl^−1^ h^−1^; for FNR1 at 1 mM in a final volume of 1 ml) were calculated based on a NADPH standard curve to determine the amount of NADPH produced in the assay (assay volume is 138 μl). Subsequently, the inverse of the rates were plotted over the inverse of the FDX concentrations in a Lineweaver–Burk plot and both *K*_m_ and *V*_max_ values were calculated for three replicates of each FDX and concentration combinations.

### EPR monitored redox titrations

Potentiometric titrations of FDX2 mutants and FDX2 for reference were carried out anaerobically in a MBraun box (N_2_ atmosphere, 25 °C) using an ORP triode electrode (internal Ag/AgCl reference, platinum sensor, Thermo Scientific 9678BNWP). The electrode was connected via a BNC cable to a pH meter (Oakton) operating in relative mV mode and calibrated to a standard solution (Orion 967901). All values were adjusted +200 mV and reported versus the normal hydrogen electrode (NHE) potential. The reaction was carried out in a custom vessel (Allen Scientific Glass, Boulder CO) with magnetic stirring using a similar reductive titration procedure as described previously (Usselman et al. 2008). NaDT was used as the reductant and added in 2 uL increments (2 mM stock) with a Hamilton repeating dispenser to the protein (2–5 mg/mL) buffer solution (50 mM Tris pH 7.8, 100 mM NaCl, 20 % glycerol). The protein buffer solution was supplemented with a redox mediator cocktail (3 μM final concentration) to allow for fast equilibrium between protein and reductant (Dutton 1978). The cocktail consisted of indigo disulfonate (*E*_m_ = −255 mV vs NHE), phenosafranine (*E*_m_ = −255 mV vs NHE), benzyl viologen (*E*_m_ = −361 mV vs NHE), and methyl viologen (*E*_m_ = −440 mV vs NHE). Samples at poised redox potentials were removed from the vessel after several minutes of equilibration following NaDT addition at roughly 20 mV increments and transferred to 4 mM EPR tubes (Wilmad LabGlass). EPR tubes were sealed with septa and frozen in liquid nitrogen.

EPR spectra were recorded on a Bruker ELEXSYS E500 CW X-band spectrometer system outfitted with an Oxford Instruments cryostat and temperature controller and cylindrical (SHQ) Bruker resonator. Spectra were collected at optimal power and temperature settings (1.0 mW, 23 ± 3 K) as determined from power saturation and temperature analysis of reduced wild-type samples (Supplemental Fig. 3). Other spectrometer settings were as follows: microwave frequency, 9.385 GHz; modulation frequency, 100 kHz; modulation amplitude, 10.0 G; and time constant, 327.68 ms. Simulations of the spectra were carried out in EasySpin (Stoll and Schweiger 2006).

To determine the midpoint potential (*E*_m_) of the [2Fe2S]^2+/1+^ cluster, plots of signal amplitude of the reduced [2Fe2S]^1+^ signal (measured at the *g* = 2.05/2.06 peak) versus sample potential (*E*) were fitted a form of the *n* = 1 Nernst equation (Hagen 2008).

$$\left[ {\text{Red}} \right] = \frac{{\left[ {\text{Ox}} \right] + [{\text{Red}}]}}{{1 + { \exp }((E - E_{\text{m}} )/0.026}}$$

Fits were carried out using the nonlinear least-squares curve fitter in OriginPro. Errors in the *E*_m_ values are estimated at ±6 mV from the standard error of the fits and voltage readouts during the experiment.

## Results

### Crystal structure of CrFDX2

**Fig. 3:**
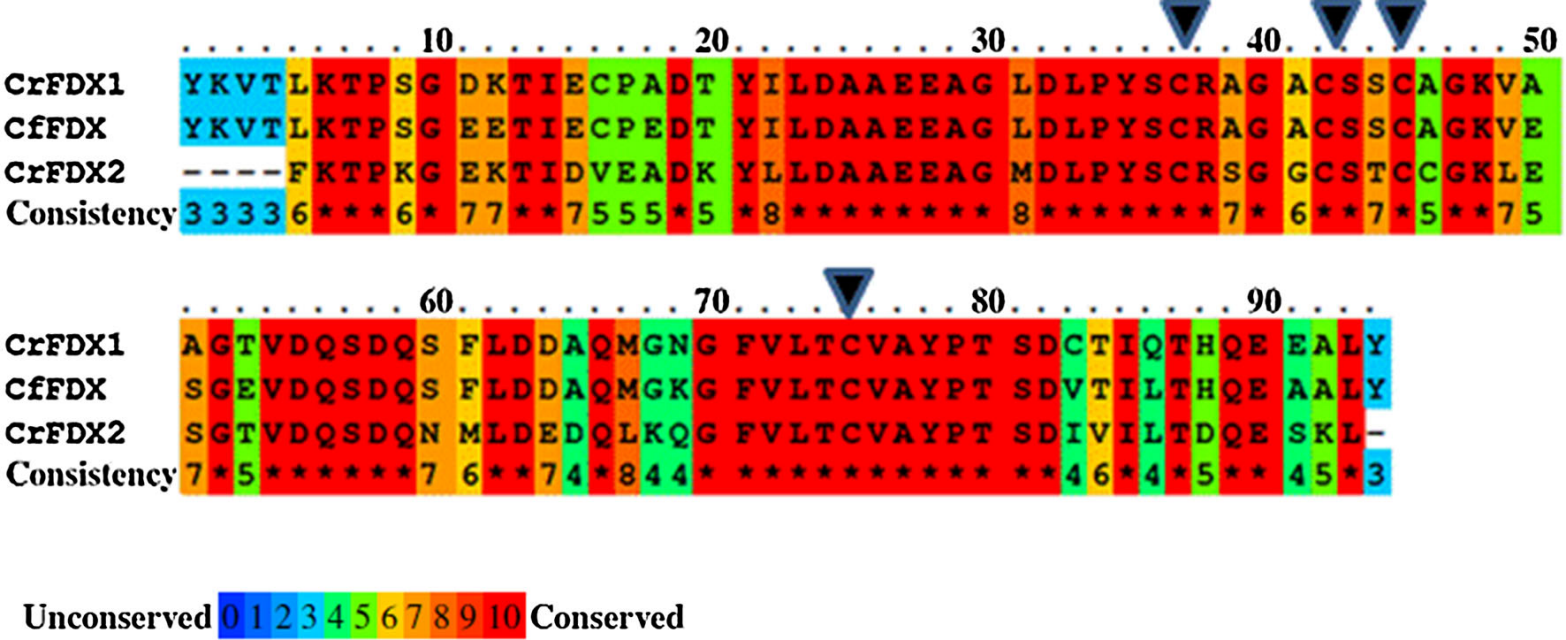
Protein sequence alignment for *Cr*FDX1, *Cr*FDX2, and *Cf* FDX. The protein sequences of the mature *Cr*FDX1, *Cr*FDX2 and *Cf*FDX proteins were aligned using the Praline multiple sequence alignment tool (http://www.ibi.vu.nl/programs/pralinewww/), and they highlight amino acid conservation among the three proteins (the three sequences show 75 % similarities). The *arrowheads* indicate the [2Fe2S]-cluster coordinating cysteine residues C37, C42, C45, and C75. All numbering differs by 1 due to the lack of the M at the start, which is present in the recombinant protein of the CrFDXs

The structure of *Cr*FDX2 was refined to a resolution of 1.18 Å with R and Rfree of 0.109 and 0.147, respectively (Fig. 1). There is only one molecule in the asymmetric unit in complex with the [2Fe2S] cluster (Fig. 1a), and it shows a typical ferredoxin fold with a β-sheet formed by five β-strands covered by a single α-helix (Fukuyama 2004). The [2Fe2S] cluster of *Cr*FDX2 is coordinated by four cysteine residues: Cys38, Cys 43, Cys 46, and Cys 76 (Fig. 2a, b; the numbering differs from that in Fig. 3 by 1, due to the lack of M at the start of each recombinant protein sequence). This structure has been deposited into the protein data bank (PDB; www.rcsb.org) with entry code 4ITK.

### Structural comparison with other FDXs

Pair-wise secondary structure matching by the PDB-fold program (Krissinel and Henrick 2004) found 60 unique structural matches for *Cr*FDX2 from the protein data bank with at least 70 % secondary structure similarity. Out of these, the first 53 were [2Fe2S]-ferredoxins and the remaining 7, although having high protein fold similarity to *Cr*FDX2, showed less than 20 % sequence similarity with it. The most similar match to *Cr*FDX2 was the cyanobacterium *Mastigocladus laminosus* [2Fe2S]-FDX (PDB ID: 1RFK; (Fish et al. 2005)), with a secondary structure similarity of 100 %, sequence similarity of 66 %, and Cα root mean square deviation of 0.84 Å^2^, suggesting similar backbones between the two proteins. Further inspections of similar ferredoxin structures showed significant variability between the positions of the backbone atoms away from the iron-sulfur cluster. This was the case even when the overall structure seemed to be highly similar. To properly find similar structures that might have been missed by the structural similarity search, we searched the PDB using sequence homology with the ICM program and found 36 structures with sequence similarity above 25 %. The best hit was *Chlorella fusca* FDX1 (*Cf*FDX1, PDB ID: 1AWD Bes et al. 1999) with sequence similarity of 68 % and Cα root mean square deviation of 0.79 Å^2^. Closer inspection of this structure showed that it indeed was highly similar to *Cr*FDX2.

### Algal CrFDX1/CrFDX2 binding interface with CrHYDA1/CrFNR1 and point mutations

The *Cr*FDX1 and *Cr*FDX2 proteins are highly homologous, with 67 % sequence identity (Fig. 3). Furthermore, *Cr*FDX2 (a root-type ferredoxin Terauchi et al. 2009) shares a practically identical protein backbone with the leaf-type *Cf*FDX1 (Fig. 3) indicating that both FDX types would be expected to share the same binding interface with *Cr*HYDA1/*Cr*FNR1 (Fig. 2a). For this interaction, we assumed that the binding interface is centered on the iron-sulfur cluster, based on a thorough analysis of related binding interfaces from literature (Chang et al. 2007; Hurley et al. 1993a, b; Morales et al. 2000). The distance between the iron-sulfur clusters is crucial for electron tunneling. According to known structures and computer modeling of electron tunneling enzyme complexes, the edge-to-edge distances should not exceed 14 Å in the absence of other additional cofactors to act as electron relays. This is the maximum distance that allows for physiologically relevant electron-transfer rates (Moser et al. 2010; Page et al. 1999; Gray and Winkler 1996). Using this information, we visually identified residues in the vicinity of the iron-sulfur cluster that were conserved between *Cr*FDX1 and *Cf*FDX1 but not in *Cr*FDX2. We specifically focused on two amino acids located near the electron acceptor site of FDX1, namely F62 and Y95: (Fig. 2b shows the location of these residues).

### In vitro hydrogen photo-production rate

To evaluate the effect of the selected mutated amino acid residues on *Cr*FDX2 biochemical properties, we measured hydrogen photo-production rates driven by *Cr*FDX1, *Cr*FDX2, and the point-mutated versions of *Cr*FDX2 (M62F, ∇95Y, and M62F/∇95Y) (see Table 2). In agreement with Peden et al. (2013), *Cr*FDX1 promoted the highest hydrogen photo-production rate, 489 (±48) μmol H_2_ μg Chl^−1^ h^−1^, while *Cr*FDX2 displayed a 5.6-fold lower rate (86 ± 11 μmol H_2_ μg Chl^−1^ h^−1^). Interestingly, each of the *Cr*FDX2 point-mutants resulted in rates that were almost twofold higher than the native *Cr*FDX2 (M62F, 150 ± 41; and ∇95Y, 134 ± 42 μmol H_2_ μg Chl^−1^ h^−1^), but lower than the *Cr*FDX1 rates. This effect appears to be additive, as the *Cr*FDX2 double mutant (M62F/∇95Y) displayed the highest rate among the three mutants (264 ± 69 μmol H_2_ μg Chl^−1^ h^−1^). The HYDA1 H_2_ evolution rate with *Cr*FDX2 M62F/∇95Y was threefold higher than with *Cr*FDX2 WT, and half of that with *Cr*FDX1. These data show that F62 and Y95 each function to support productive electron-transfer complexes between *Cr*FDX1 and *Cr*HYDA1 and, when engineered onto *Cr*FDX2, confer it higher catalytic rates. In other words, mutations that introduce these *Cr*FDX1 amino acid residues onto *Cr*FDX2 are likely to induce similar structural changes in *Cr*FDX2 to promote higher hydrogen production rates, closer to those measured with *Cr*HYDA1.

**Table 2:** Hydrogen photo-production rates

Ferredoxin	Hydrogen Photo-production Rate (μmol H_2_ μg Chl^−1^ h^−1^)
*Cr*FDX1	489 ± 48
*Cr*FDX2	86 ± 11
*Cr*FDX2 (M62F)	150 ± 41
*Cr*FDX2 (∇95Y)	134 ± 42
*Cr*FDX2 (M62F/∇95Y)	264 ± 69

The *Cr*FDX1, *Cr*FDX2, and mutated *Cr*FDX2 proteins used for the hydrogen photo-production assay were the cleaved forms purified from proteins over-expressed in *E. coli* from the respective FDX-TEVcs-GST-His constructs. Individual rates were calculated from four time points (approximately *t* = 60, 180, 300 and 420 min) and the averaged rates are shown

### In vitro NADPH photo-production kinetic parameters

The kinetic parameters *K*_m_ (μM), *V*_max_ (μmol NADPH μg Chl^−1^ h^−1^) and *k*_*cat*_ (s^−1^), of *Cr*FDX1, *Cr*FDX2, and the *Cr*FDX2 point-mutants for the NADPH photo-production reaction were determined from the respective Lineweaver–Burk plots (see Table 3 and Supplemental Fig. 5). Interestingly, the *V*_max_ values for NADPH photo-production were similar for *Cr*FDX1 and *Cr*FDX2, at 185 (±68) and 177 (±47) μmol NADPH μg Chl^−1^ h^−1^, respectively. However, the *K*_m_ for *Cr*FDX2 (0.18 ± 0.01, μM) was significantly lower than that for *Cr*FDX1 (0.40 ± 0.04, μM), indicating *Cr*FDX2′s higher affinity for *Cr*FNR1. On the other hand, *K*_m_ values for each of the *Cr*FDX2 mutants were higher than that for the native *Cr*FDX2 (Table 3), with values of 0.69 ± 0.04 μM for the M62F mutant, 0.38 ± 0.03 μM for the ∇95Y mutant, and 3.44 ± 0.71 μM for the M62F/∇95Y double mutant. The calculated *V*_max_ values for the FNR-catalyzed NADPH production by the mutant proteins were 161 ± 26 μmol NADPH μg Chl^−1^ h^−1^for the M62F mutant, 202 ± 10 μmol NADPH μg Chl^−1^ h^−1^ for the ∇95Y mutant, and 260 ± 33 μmol NADPH μg Chl^−1^ h^−1^ for the M62F/∇95Y double mutant. These values are very similar to those measured with either *Cr*FDX1 or *Cr*FDX2 WT proteins, considering the error bars. The catalytic efficiencies of *Cr*FDX2 and *Cr*FDX2 mutants in driving the *Cr*FNR1-dependent reaction, *k*_cat_/*K*_m_, showed interesting trends compared to *Cr*FDX1 (Table 3). Although *Cr*FDX2 supported a slightly lower *V*_max_, the lower *K*_m_ with *Cr*FNR1 resulted in a twofold larger *k*_cat_/*K*_m_, indicating the formation of a more efficient catalytic complex. On the other hand, *Cr*FDX2 M62F presented a fourfold lower *k*_cat_/*K*_m_ compared to *Cr*FDX2 and twofold lower than *Cr*FDX1. The *Cr*FDX2 ∇95Y variant led to a slightly higher *V*_max_ but lower *k*_cat_/*K*_m_, a value that is more similar to that measured with *Cr*FDX1. When the M62F mutation was paired with ∇95Y in the *Cr*FDX2 double mutant, the resulting variant supported the highest *k*_cat_, but led to a large, 13-fold decrease in *k*_cat_*/K*_m_ compared to *Cr*FDX2. Overall, the kinetics suggest that *Cr*FDX2, under growth conditions where it is present in equimolar amounts to *Cr*FDX1, is better at electron transfer with FNR1 than *Cr*FDX1, by virtue of forming a more efficient catalytic complex with FNR1. In contrast, *Cr*FDX1 catalyzes higher rates of H_2_ evolution, and the changes (although small) observed with the M62F and ∇95Y mutants of *Cr*FDX2 are evidence for involvement of these residues in H_2_ evolution.

**Table 3:** Kinetic values for FDX-mediated NADPH photo-production in a reconstituted system

Ferredoxin	*K*_m_ NADP^+^ (µM)	*V*_max_ NADP^+^ (μmol NADPH μg Chl^−1^ h^−1^)	Turnover *k*_cat_ (s^−1^) for NADP^+^ reduction	Efficiency *k*_cat_/*K*_m_ (M^−1^ s^−1^) for NADP^+^ reduction
*Cr*FDX1	0.40 ± 0.04	185 ± 68	668 ± 25	17 × 10^8^
*Cr*FDX2	0.18 ± 0.01	177 ± 47	638 ± 17	35 × 10^8^
*Cr*FDX2 (M62F)	0.69 ± 0.04	161 ± 26	581 ± 9	8.4 × 10^8^
*Cr*FDX2 (∇95Y)	0.38 ± 0.03	202 ± 10	729 ± 37	19 × 10^8^
*Cr*FDX2 (M62F/∇95Y)	3.44 ± 0.71	260 ± 33	939 ± 120	2.7 × 10^8^

The FDX1, FDX2, and mutated FDX2 proteins used for the NADPH photo-production assay were prepared by TEV treatment of FDX-TEVcs-GST-His fusions. Both *K*_m_ and *V*_max_ values were calculated from three independent replicates using linear regression analyses of Lineweaver–Burk plots. Supplemental Fig. 5 shows the Lineweaver–Burk plots for the averaged rates. NADPH photo-production rates (*V*_max_) are given in μmol NADPH μg Chl^−1^ h^−1^ with 1 mM FNR1 present and a 1-ml assay volume were calculated from the initial 15 min after mixing. The *k*_cat_ values were calculated at FDX concentrations of 1 mM

### Midpoint redox potential determination

**Fig. 4:**
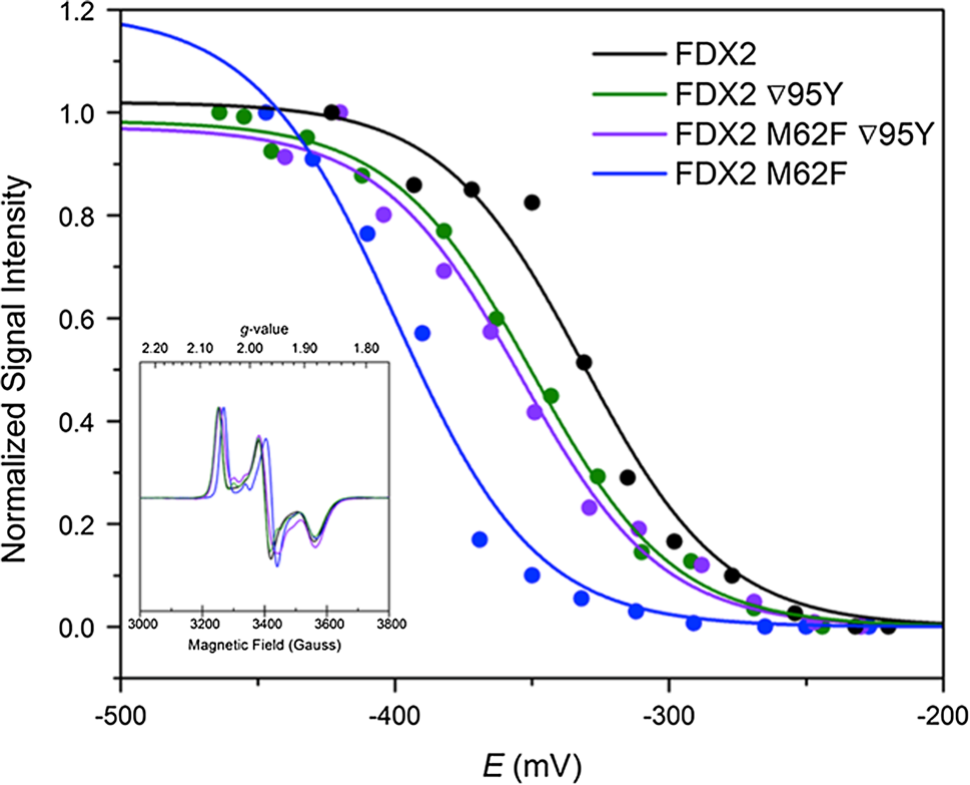
Redox titrations of the [2Fe2S]-EPR signal (*inset*) from reduced *Cr*FDX2 wild type, ∇95Y, M62F/∇95Y, and M62F mutants. Each point reflects a measured EPR amplitude of the *g* = 2.05/2.06 peak for individual samples poised at particular potentials (*E*) vs NHE. The midpoint potentials were determined by fitting the potentiometric curves to the *n* = 1 electron form of the Nernst Equation

The midpoint redox potentials of the *Cr*FDX2 mutants were determined to test the effect of the mutations on the electron transfer properties of the [2Fe2S] cluster. Both of the single mutations shifted the potential more negative compared to *Cr*FDX2, with the M62F mutation giving the largest shift bringing it close to the midpoint potential of *Cr*FDX1 (Fig. 4, Table 4). Surprisingly, the double mutant did not show an additive shift of the single mutations but rather displayed a redox potential similar to that of the ∇95Y alone. While the overall rhombic signal assigned to the [2Fe2S] cluster was almost identical in all cases, slight shifts in the *g*-values were observed for the mutants. It should be noted that appearance of other small signals were observed; however, these are likely from the redox cocktail and formation of radical species during the course of reduction with NaDT. The shifts in the rhombic [2Fe2S]-cluster signal can be summarized by a subtle upfield energy shift from the *g*-values of *Cr*FDX2 (*g* = 2.06, 1.97, and 1.88) to the *g*-values of *Cr*FDX1 (*g* = 2.05, 1.96, and 1.88). Compared to ∇95Y (*g* = 2.063, 1.973, and 1.883) and M62F/∇95Y (*g* = 2.060, 1.969, and 1.883), M62 showed the largest shift (*g* = 2.052, 1.959, and 1.880) resulting in its overall signal to more closely align with *Cr*FDX1 (Fig. 4 inset). Interestingly, these trends match nicely to the midpoint potential shifts of the mutants and particularly for M62F may indicate an underlying role toward finely tuning the orientation and electronic properties of the cluster.

**Table 4:** The midpoint potentials of FDX2 mutants compared to *Cr*FDX1 and *Cr*FDX2 wild types

Ferredoxin	*E*_m_ (vs NHE)
*Cr*FDX1	−398^a^
*Cr*FDX2	−331^b^ (−321^c^)
*Cr*FDX2 (M62F)	−400
*Cr*FDX2 (∇95Y)	−350
*Cr*FDX2 (M62F/∇95Y)	−356

^a^Ref (Galván and Márquez 1985)

^b^This study

^c^Ref. (Terauchi et al. 2009)

## Discussion

Recently, we initiated efforts to fully characterize the Chlamydomonas FDX interaction network (Peden et al. 2013). We and others had shown previously that *Cr*FDX1 plays a predominant role as an electron carrier in the cell, through electron-transfer and binding interactions with multiple partners (Noth et al. 2012; Peden et al. 2013; Terauchi et al. 2009; van Lis et al. 2013). Interestingly, *Cr*FDX2 was demonstrated to be capable of binding in vitro to some of the same electron partners and promoting similar redox reactions as *Cr*FDX1 (Noth et al. 2012; Peden et al. 2013; van Lis et al. 2013).

In order to determine and compare the characteristics and functions of the two *Cr*FDXs in more details, and to identify and study the nature of their interaction with other enzymes, we performed additional biochemical and biophysical assays on the two-purified proteins. The spectroscopy studies confirmed that both proteins are highly similar, showing typical [2Fe2S]-ferredoxin spectra (Supplemental Figs. 2, 3, and 4). We also grew crystals and solved the *Cr*FDX2 structure, which represents the first solved Chlamydomonas FDX structure. A structure of *Cr*FDX1 from *Chlorella fusca* was previously reported (Bes et al. 1999), and it was used for the visual identification of differences between *Cr*FDX1 and *Cr*FDX2. Five different amino acids located in the vicinity of the [2Fe2S] cluster and at the binding interface between both *Cr*FDXs and *Cr*HYDA1 were identified (Winkler et al. 2009a). As such, they have the potential to affect the binding properties of either of the two *Cr*FDXs, or possibly their electron transfer potentials to specific donors/acceptors. We mutated two residues in *Cr*FDX2 to resemble those present in *Cr*FDX1 and determined the effects on two reactions: NADP^+^ reduction and H_2_ photo-production.

The catalytic properties of each of our FDX mutants are different with respect to electron transport to *Cr*HYDA1 and *Cr*FNR1, as shown in Tables 2 and 3, respectively. We demonstrated that introduction of Y95 and mutation of *Cr*FDX2 M62 to phenylalanine directly affects its hydrogen and NADPH photo-production activity. The single *Cr*FDX2 M62F and ∇95Y mutants showed higher hydrogen photo-production rates than the *Cr*FDX2 WT protein, but not as high as that of *Cr*FDX1. This demonstrates that both residues in *Cr*FDX1 promote high hydrogen production, either by affecting protein complex formation/stabilization, electron transfer, or both. This was to be expected from the structural results that showed the proximity of these residues to the *Cr*FDX: *Cr*HYDA1 binding interface (Fig. 2b). Interestingly, the double mutant was able to support hydrogen production at an even higher rate than either of the two single mutants and, in fact, twofold higher than the *Cr*FDX2 WT (Table 2). The additive effect of these mutations suggests that both residues in *Cr*FDX1 contribute to its high H_2_ production. Indeed, both mutations resulted in shifts in the midpoint redox potentials of *Cr*FDX2 toward more negative values (Table 4), which favor electron transfer to *Cr*HYDA1.

We also tested the kinetics of WT and point-mutated *Cr*FDX2 proteins in NADPH photo-production. We show that the presence of *Cr*FDX1 F62 and Y95 in *Cr*FDX2 affect NADPH production in opposite manners. The presence of F62 in *Cr*FDX2 interferes mostly with its binding affinity to *Cr*FNR (higher *K*_m_ compared to wild-type FDX2) and leads to a decrease in the turnover of NADPH production (*k*_cat_). This could be due to the direct interaction between residue 62 and *Cr*FNR’s FAD co-factor (Supplementary Fig. 4c, e), which may be weakened by the presence of methionine in this position. On the other hand, the deletion of Y95 from *Cr*FDX2 affects all kinetic parameters of the FNR-mediated reaction, as shown by an increase in its *K*_m_, K_cat_ and catalytic efficiency (levels similar to those obtained with *Cr*FDX1) (Table 3). These residues are therefore important for the *Cr*FDX1: *Cr*FNR interaction and photo-reduction of NADP^+^. It must be noted that others had shown that the *Cr*FNR has almost identical *k*_cat_*s* in the presence of either *Cr*FDX1 or *Cr*FDX2, but the *K*_m_ for *Cr*FDX2 was almost sixfold lower than that for *Cr*FDX1, resulting in a sixfold higher catalytic efficiency for *Cr*FDX2 (Hurley et al. 1997; Vieira and Davis 1986). However, the reported values were derived from an assay that indirectly measured electron transfer between ferredoxin and *Cr*FNR using cytochrome c reductase activity; therefore their results are not directly comparable to ours.

The kinetics parameters observed for the *Cr*FDX2 double mutant seem to be a combined effect of the two single mutations, yielding a protein with a much higher *K*_m_ (as the single M62F mutant), but also high *V*_max_ (high rates of NADP reduction, as the single ∇95Y mutant) and high *k*_cat_ (higher catalytic efficiency, as the single ∇95Y mutant), although low catalytic efficiency. These differences are accompanied by structural differences at positions 62 and 95 in the *Cr*FDXs, which could partially account for the observed differences in kinetics. These observations are also consistent with the concept that subtle changes involving this particular structural region have significant effects in electron transfer within the functional catalytic complex. Indeed, all the mutations introduced at position 62 and 95 of *Cr*FDX2, singly or in combination led to changes in redox-midpoint potentials as compared to *Cr*FDX2.The EPR titration data (Table 4) showed that the *E*_m_ value of the ∇95Y *Cr*FDX2 mutant shifted more negatively, closer to the *Cr*FDX1 value (−350 vs −398 vs −331 mV for ∇95Y, *Cr*FDX1 and *Cr*FDX2, respectively). The double mutant M62F/∇95Y showed a similar pattern with an *E*_*m*_ of −350 mV where the single M62F mutant had midpoint potential of −400 mV almost identical to *Cr*FDX1 (−398 mV Terauchi et al. 2009).

In previous literature, certain amino acid residues were shown to be important specifically for the *Cr*FDX1/*Cr*HYDA1 interaction, and to be critical for efficient electron transfer between these two proteins. *In silico* docking analysis and site-directed mutagenesis, for instance, identified (among ten amino acid residues tested) *Cr*HYDA1 K396, and FDX1 E122 (amino acid numbers represent the position in the protein prior to cleavage of the transit peptide) as the major contributors to the formation of the FDX-HYD1 complex (Winkler et al. 2010). Interestingly, the two *Cr*HYDA1 and *Cr*HYDA2 share conservation of the required lysine, and five *Cr*FDXs (except for *Cr*FDX3) contain the conserved glutamic acid residue. Residues D56 and F93 in FDX1 were also shown to be important for the *Cr*FDX1-*Cr*HYDA1 interaction; F93 together with E122 and Y126 was proposed to be involved in stabilizing the redox state of [2Fe2S] cluster of *Cr*FDX1, suggesting their probable role in electron transfer between *Cr*FDX1 and *Cr*HYDA1 (Winkler et al. 2010). Indeed, when mutated to nonconserved residues, the respective recombinant proteins showed a decreased *V*_max_ for H_2_ photo-production of more than threefold compared to the WT value (11).

Electrostatic interactions have also been demonstrated to be crucial for the interaction between ferredoxins and all their other target enzymes, such as FNR, FD:thioredoxins reductase, nitrite reductase, glutamate synthase, and sulfite reductase (Hanke and Mulo 2013). More specifically, *Cr*FDX1 interaction-complex studies have provided evidence for the essential role of another conserved glutamate, E91, located in the short C-terminal tail of *Cr*FDX1, and of a negatively charged patch, located in its most N-terminal α1-helix, which includes D25, E28, and E29. All four negatively charged residues are conserved only in *Cr*FDX1, *Cr*FDX2, and *Cr*FDX5, out of the six *Cr*FDXs (Terauchi et al. 2009). E91 is involved in forming complexes with nitrite reductase (NiR), glutamate synthetase (FDX-GOGAT), and the photosystem I subunit C (PSAC). Mutations of this residue in *Cr*FDX1 diminish its catalytic activity in the reactions involving these enzymes (Fischer et al. 1998; GarciaSanchez et al. 1997). In addition, a triple D25A/E28Q/E29Q mutant protein showed less efficient interaction with those same three interacting enzymes (GarciaSanchez et al. 1997; Jacquot et al. 1997).

Besides electrostatic interactions, the midpoint redox potential of the two *Cr*FDXs plays an important role in their physiological functions. Normally, *Cr*FDX2 catalyzes nitrite reduction (Terauchi et al. 2009), a reaction with a midpoint redox potential of about −300 mV; NADP^+^ reduction and H_2_ production, on the other hand, are catalyzed by *Cr*FDX1 in vivo, in reactions that require more negative redox potentials (−320 and −400 mV, respectively). It was shown that the presence of an aromatic residue at position 65 in Anabaena ferredoxin is essential for effective electron transfer with FNR (Hurley, cheng et al. 1993), and that natural variants of animal and bacterial FDXs in which methionine is replaced by phenylalanine show a shift in the *E*_m_ to more positive values (Hurley et al. 1993a, b, 1997). We suspected that the switch from phenylalanine at residue 62 in *Cr*FDX1 to methionine in *Cr*FDX2 (Fig. 2b), in particular, was responsible for the significantly different *Cr*FDX2 ability to catalyze NADPH and H_2_ production. In this study, we measured and compared redox potential and kinetic parameters for the NADPH and H_2_ photo-production between the *Cr*FDX2 and the *Cr*FDX2 M62F and ∇95Y mutants with those of *Cr*FDX1. NADPH production, which involves NADP^+^ + 2H^+^ binding, involves a more complex reaction, since it requires the binding of both NADP^+^ and 2H^+^ to the FDX/FNR complex when compared to the FDX/HYDA interaction which requires only the binding of 2H^+^. Finally, although our *Cr*FDX2 crystal structure shows that both residues are located near the FDX [2Fe2S] cluster and could therefore influence the catalytic activity of FDX when in complex with FNR/HYDA, it seems that this is in fact organism dependent. In Anabaena, for example, FDX undergoes a conformational change at the level of the loop that contains F65 (the equivalent of F62 in Chlamydomonas) upon binding to FNR (Morales et al. 2000). Furthermore, this amino acid is proposed to be involved in the electronic coupling between the two redox centers (Hurley et al. 1993a). On the other hand, the maize leaf FDX/FNR crystal structure complex revealed that the equivalent amino acid of F65 (here Tyr 63) is neither in close contact with the [2Fe2S] cluster of the FDX, nor is between the two prosthetic groups from FNR (FAD) and FDX ([2Fe2S]) suggesting a different role in electron transfer to FNR for that residue (Kurisu et al. 2001). Unfortunately we do not have a crystal structure of the Chlamydomonas FDX/FNR complex to verify either mechanism. An alternative possible explanation for our kinetic data is that the mutations could interfere with PSI binding, which would further affect electron transfer to the FDXs. The data could therefore indicate that the mutations perturb the conformation of the *Cr*PSI: *Cr*FDX2 complex solely or in addition to the *Cr*FDX2:*Cr*HYDA1 complex. It is known that PSI subunits interact with *Cr*FDX, although it remains unclear how many sites are present and/or available for FDX binding on *Cr*PSI. Furthermore, no information regarding the *K*_m_ for *Cr*PSI: *Cr* FDX electron transfer is available, although *K*_d_ values for WT and some PSI mutants ranging from 6 to 0.12 µM have been reported (Setif 2001; Setif et al. 2002). Finally, it must be noted that the error bars reflecting the double mutant data were particularly high, possibly due to the higher instability of the double mutant protein. The actual kinetic values must thus be taken only as representing a trend, not an actual number.

In summary, despite their high sequence similarity and comparable physical characteristics, *Cr*FDX1 and *Cr*FDX2 also exhibit structural differences that affect their electron-transfer function. A previous structural model predicted differences in the surface charge distribution between the two proteins (Terauchi et al. 2009), and our *Cr*FDX2 structure and biochemical results further show that the *Cr*FDX2 M62 and *Cr*FDX1 95Y residues make significant contributions to the binding interfaces of the respective *Cr*FDXs with *Cr*HYDA1 and *Cr*FNR1, as well as affecting their redox potentials. These distinct differences must certainly contribute to the different in vivo specificities of the two proteins (Gou et al. 2006; Terauchi et al. 2009). In this report, we indirectly demonstrate that the *Cr*FDX1 F62 and Y95 residues are important for hydrogen photo-production, as progressively increased hydrogen production rates are measured when these residues are introduced into *Cr*FDX2. Residues F62 and Y95 also affect NADPH photo-production and have opposite impacts on the kinetic parameters of that reactions. We thus confirm that *Cr*FDX2 can potentially replace *Cr*FDX1 in *Cr*FDX1-dependent reactions and that differences between the two proteins rely on differences between only a few amino acid residues.

## Electronic supplementary material

Supplementary material 1 (DOCX 542 kb)

## Abbreviations

CD: Circular dichroism
DCMU: (3-(3,4-Dichlorophenyl)-1,1-dimethylurea)
DCPIP: 2,6-Dichlorophenolindophenol
DMSO: Dimethyl sulfoxide
DTT or NaDT: Sodium dithionite
EPR: Electron paramagnetic resonance
FAD: Flavin adenine nucleotide
FDX: Ferredoxin
FNR: Ferredoxin/NADP(H) oxidoreductase
FPLC: Fast protein liquid chromatography
GST: Glutathione S-transferase
HYDA: Algal hydrogenase
NADP(H): Nicotinamide adenine dinucleotide phosphate-oxidase
PCR: Polymerase chain reaction
PFR1: Pyruvate/ferredoxin oxidoreductasePDB, protein database
RT: Room temperature
TEV: Tobacco etch virus protease
