# Supplementary material for: Crystal structure and biochemical characterization of Chlamydomonas FDX2 reveal two residues that, when mutated, partially confer FDX2 the redox potential and catalytic properties of FDX1
Source: Photosynth Res. 2015 Nov 3;128:45–57. doi: 10.1007/s11120-015-0198-6 (PMC4791469; doi:10.1007/s11120-015-0198-6)
Supplement: Supplementary file 1 — Supplementary material 1 (DOCX 542 kb) [file 11120_2015_198_MOESM1_ESM.docx]

Supplemental Figures

*FDX expression and Purification*

In order to investigate the basic spectroscopic properties of *Cr*FDX1 and *Cr*FDX2, both proteins (and *Cr*FDX2 mutants) were purified as described in Material and Methods. Fractions for SDS-PAGE analysis were taken during the process (Supplemental Figure 1). For these studies, the proteins were expressed using the His-GST-TEVcs-FDX and FDX-TEVcs-GST-His constructs, yielding approximately 5 mg of each purified recombinant protein per L of *E. coli* cell culture. In all cases, the final samples contained no apparent contaminations and the molar ratio of iron per protein was 2.93 (+/- 0.13) and 2.24 (+/- 0.05) for the cleaved forms of *Cr*FDX1 and *Cr*FDX2, respectively. Further analysis by UV-vis (Supplemental Figure 2), EPR (Supplemental Figure 3), and CD spectroscopy (Supplemental Figure 4) revealed similar signals in all cases typical for [2Fe2S]-cluster FDXs, and no major differences between the two FDXs were observed.

Figure 1


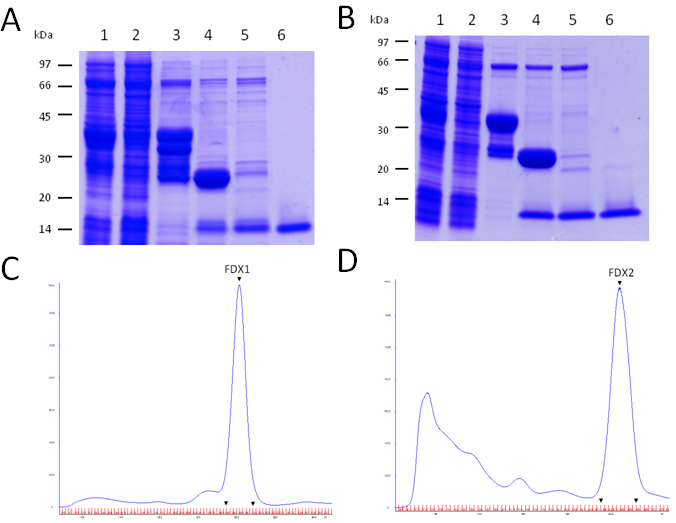


**SDS-PAGE analysis of the fractions collected** **for *Cr*FDX1 and *Cr*FDX2 during purifications.** (A) *Cr*FDX1 (using the pRSETA His-GST-TEVcs-FDX1 expression construct) and (B) *Cr*FDX2 (using the pRSETA FDX2-TEVcs-GST-His expression construct): 1 cell lysate, 2 flow through from the GST column, 3 elution pool from the GST column, 4 sample after TEV cleavage, 5 flow through from a TALON Co-resin column and 6 sample after size exclusion chromatography. The two proteins molecular weight is just under 14kD.

Figure 2


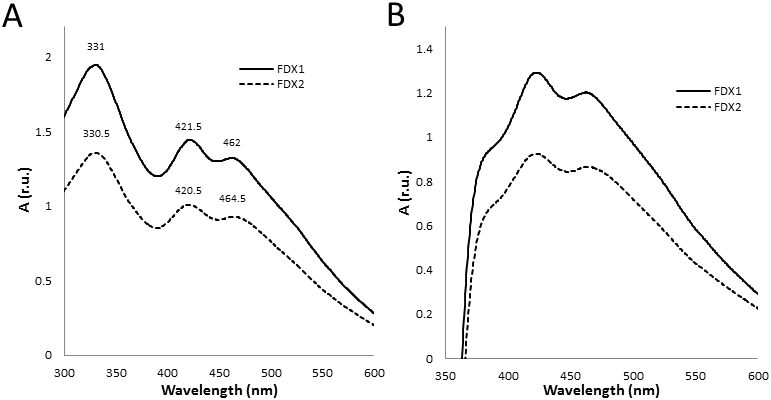


**421.5**

**462**

**420.5**

**464.5**

**UV/Vis spectra of *Cr*FDX1 and *Cr*FDX2.** The *Cr*FDX1 and *Cr*FDX2 proteins used in this experiment were overexpressed in *E. coli* using the His-GST-TEVcs-FDX expression constructs, purified, and the TEV tag cleaved, as described in Materials and Methods. (A) The UV/Vis spectra of oxidized (as is) *Cr*FDX1 and *Cr*FDX2 (approximate protein concentrations of 1 mg ml^-1^). (B) The oxidized (as is) minus sodium dithionite-reduced (16 mM NaDT) difference spectra for *Cr*FDX1 and *Cr*FDX2. The UV/Vis spectra of the oxidized proteins (as is) displayed typical ferredoxin absorption maxima at 331, 421.5 and 462 nm for *Cr*FDX1 and at 330.5, 420.5 and 464.5 nm for *Cr*FDX2 (Figure 1A; ([Schmitter *et al.*, 1988](#_ENREF_43)). Upon reduction with 16 mM NaDT, the peaks in the 420 and 460 nm region disappeared, as shown in the oxidized-minus-reduced absorption difference spectra (Figure 1B). The A420/A275 ratios were calculated to be 0.59 and 0.65 for *Cr*FDX1 and *Cr*FDX2, respectively, indicating highly purified ferredoxin preparations ([Ashton & Anderson, 1981](#_ENREF_1)).

Figure 3


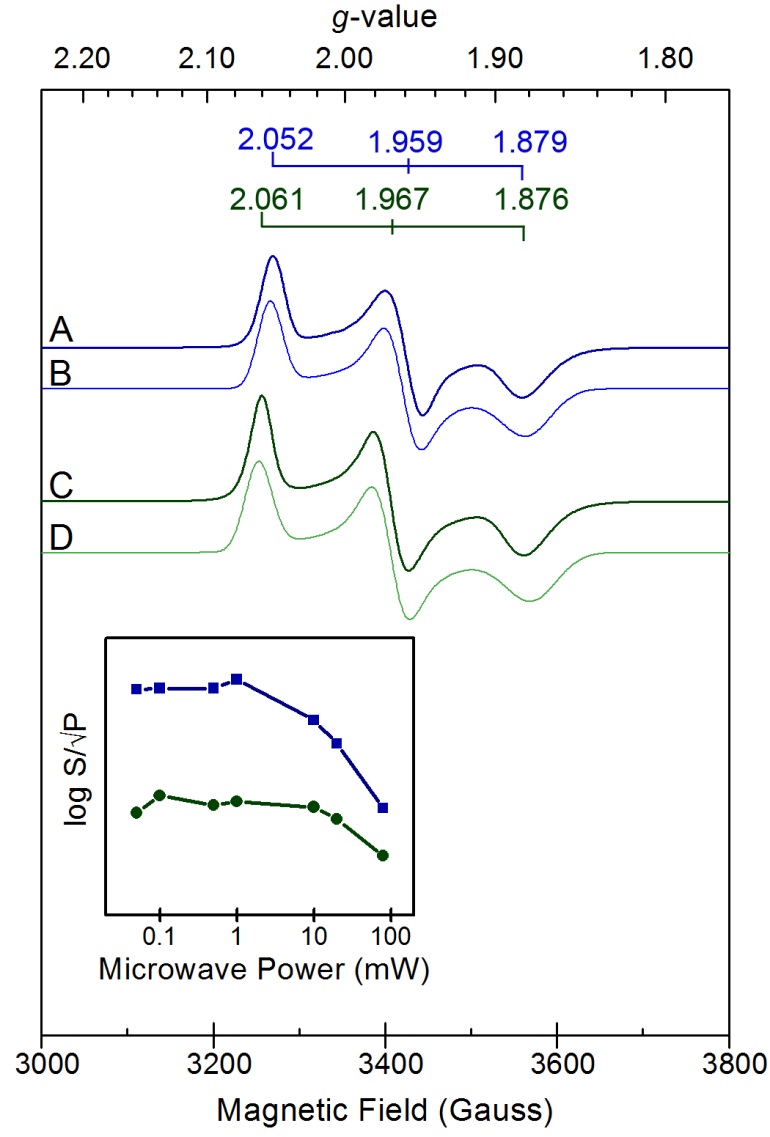


**EPR spectra and power saturation curves of reduced *Cr*FDX1 and *Cr*FDX2.** (A) *Cr*FDX1 (250 µM) reduced with 20 mM NaDT and (B) simulation (*g* = 2.052, 1.959, 1.879). (C) *Cr*FDX2 (250 µM) reduced with 20 mM NaDT and (D) simulation (*g* = 2.061, 1.967, 1.876). Overall, both signals are typical of [2Fe2S]-cluster signals and similar to previous reports of natively expressed plant-type ferredoxins (Galván & Márquez, 1985[Galván & Márquez, 1985](#_ENREF_12), [Hall *et al.*, 1973](#_ENREF_17)). The inset shows the microwave power dependence of the *Cr*FDX1 (◼) and *Cr*FDX2 (●) signals as measured by the spectral features at *g* = 2.052 and *g* = 2.061, respectively, at 23 K (S, signal amplitude; P, power). Both signals displayed just small degrees of power saturation broadening. The *Cr*FDX1 signal was more sensitive to saturation broadening indicating slightly longer relaxation times compared to *Cr*FDX2. Spectrometer settings: temperature, 23 K; microwave power, 1.0 mW, microwave frequency, 9.385 GHz; modulation frequency, 100 kHz; modulation amplitude, 10.0 G; time constant, 327.68 ms.

Figure 4


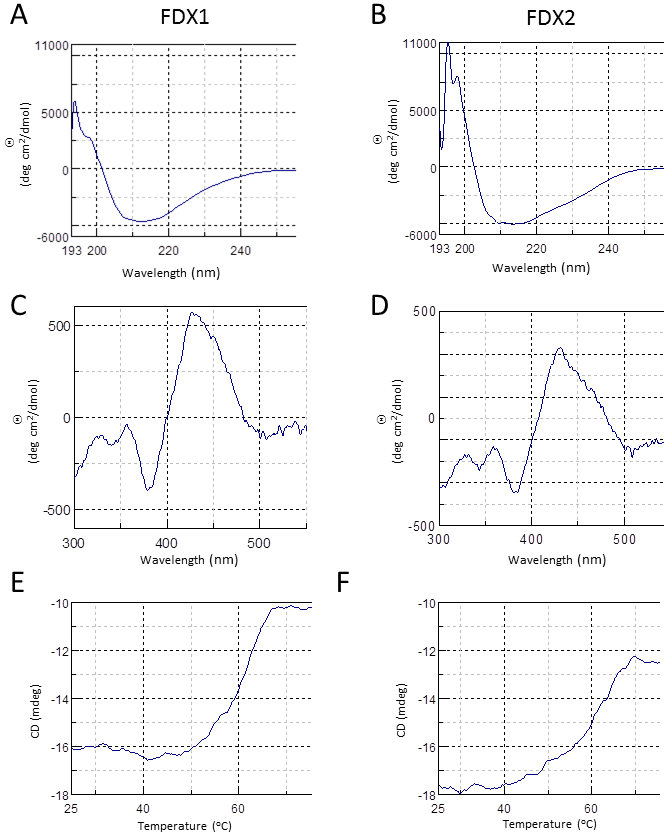


**CD spectroscopy of *Cr*FDX1 and *Cr*FDX2.** The *Cr*FDX1 and *Cr*FDX2 proteins used in this experiment were overexpressed in *E. coli* using the FDX-TEVcs-GST-His expression constructs, purified, and the TEV tag cleaved, as described in Materials and Methods. (A and B) CD spectra in the near UV region (190 to 260 nm) (C and D) CD spectra between 300 and 600 nm detecting the [2Fe2S] chromophore and (E and F) melting curves monitored at 222 nm to determine thermal stability.

Figure 5


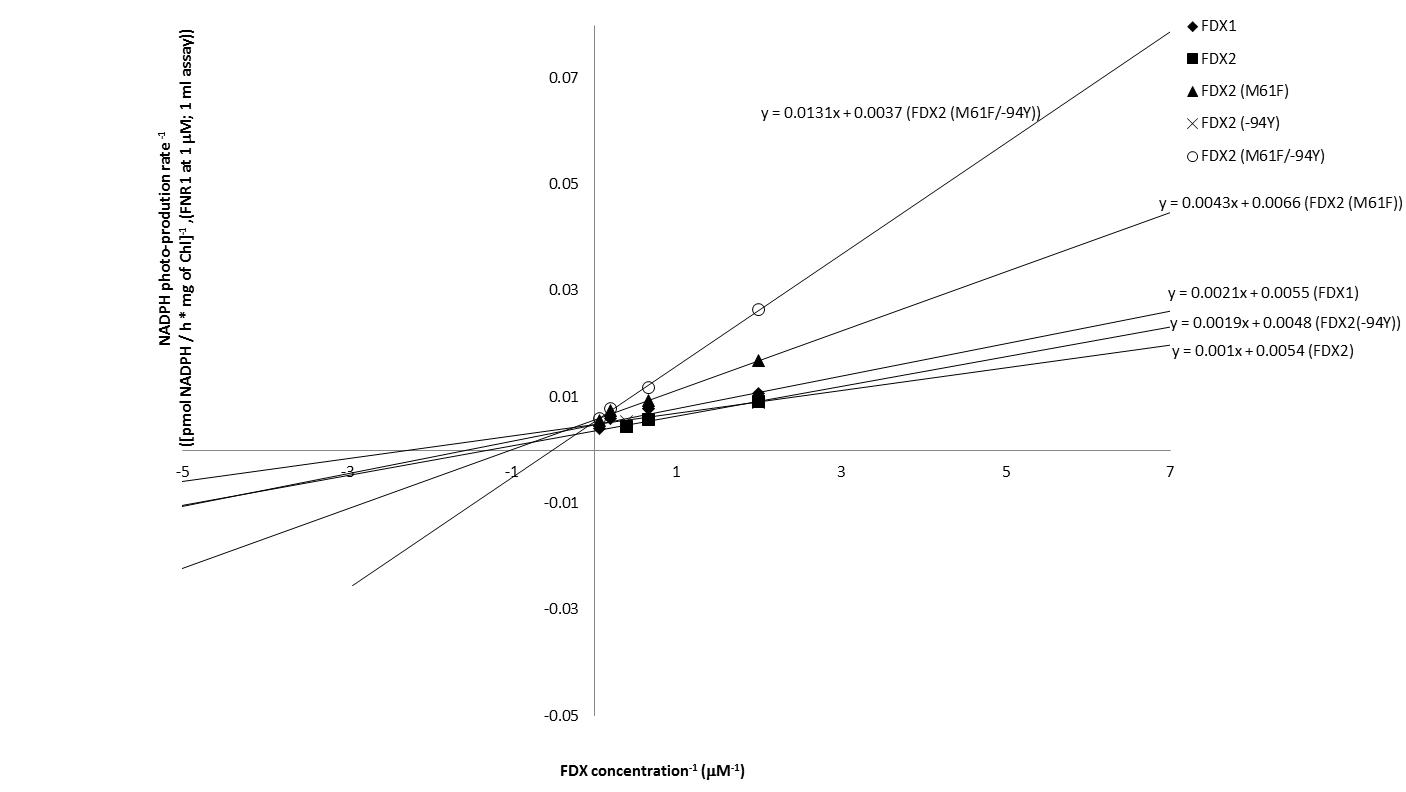


**Lineweaver-Burke plot for FDX-mediated NADPH photoproduction.** The average rates from three independent replicates at each FDX concentration were used to generate the plot. For the sake of clarity, error bars are not represented.
